# Fine Structure and Olfactory Reception of the Labial Palps of *Spodoptera frugiperda*

**DOI:** 10.3389/fphys.2021.680697

**Published:** 2021-08-03

**Authors:** Qiuyan Chen, Xiaolan Liu, Song Cao, Baiwei Ma, Mengbo Guo, Jie Shen, Guirong Wang

**Affiliations:** ^1^State Key Laboratory for Biology of Plant Diseases and Insect Pests, Institute of Plant Protection, Chinese Academy of Agricultural Sciences, Beijing, China; ^2^Department of Entomology and MOA Key Laboratory for Monitory and Green Control of Crop Pest, China Agricultural University, Beijing, China; ^3^Shenzhen Branch, Guangdong Laboratory for Lingnan Modern Agriculture, Genome Analysis Laboratory of the Ministry of Agriculture and Rural Affairs, Agricultural Genomics Institute at Shenzhen, Chinese Academy of Agricultural Sciences, Shenzhen, China

**Keywords:** *Spodoptera frugiperda*, olfactory, labial-palp pit organ, CO2, volatiles

## Abstract

The olfactory system of insects is essential in many crucial behaviors, such as host seeking, mate recognition, and locating oviposition sites. Lepidopteran moths possess two main olfactory organs, including antennae and labial palps. Compared to antennae, the labial palps are relatively specific and worthy of further investigation due to the labial-palp pit organ (LPO), which contains a large number of sensilla located on the tip segment. The fall armyworm, *Spodoptera frugiperda*, is a worldwide lepidopteran pest, which can damage more than 350 plants and cause significant economic losses. In this study, we surveyed the structure of the labial palps and LPO of *S. frugiperda* using a super-high magnification lens zoom 3D microscope. Then, the distribution and fine structure of sensilla located in the LPO of *S. frugiperda* were investigated using scanning electron microscopy. Subsequently, the electrophysiological responses of labial palps to CO_2_ and 29 plant volatiles were recorded by using electrolabialpalpography. Our results showed the fine structure of labial palps, the LPO, and the sensilla located in the LPO of *S. frugiperda*. Moreover, we demonstrated that the labial palps are olfactory organs that respond to both CO_2_ and other volatile compounds. Our work established a foundation for further study of the roles of labial palps in insect olfactory related behaviors. Further investigations on the function of labial palps and their biological roles together with CO_2_ and volatile compound responses in *S. frugiperda* are necessary, as they may provide better insect behavioral regulators for controlling this pest.

## Introduction

The sophisticated olfactory sensing organs of most insects have important roles in detecting host volatiles, recognizing mates, and locating oviposition sites. These organs are mainly distributed in the head, including antennae and mouthpart appendages. As the primary olfactory sensory organs, insect antennae bear abundant of sensilla that are sensitive to plant volatiles, sex pheromones, and other volatile components. Additionally, some olfactory sensilla are also found on mouthpart appendages, such as maxillary palps (Syed and Leal, 2007; Bohbot et al., 2014) and labial palps (Stange and Stowe, 1999; Galizia and Rossler, 2010). As an important sensory organ, the well-developed labial palps are located on each side of the proboscis in adult Lepidoptera. The labial-palp pit organ (LPO) is a unique structure of lepidopteran species that is located on the apex of labial palps, within which the sensilla lie.

The labial palps are densely covered with scales and usually contain three segments. If the scales are removed, a bottle-shaped LPO situated on the tip of the third segment of the labial palp can be observed. Detailed electron microscopical analyses have been performed on the structure of the LPO in many lepidopteran species (Stange and Stowe, 1999; Faucheux, 2008; Zhao et al., 2013; Dong et al., 2014; Barcaba and Krenn, 2015; Chen and Hua, 2016; Yan et al., 2019), which not only showed large numbers of olfactory sensilla in the LPO but also provided descriptions of the fine structure of LPO and LPO sensilla. The morphological characteristics of LPO and LPO sensilla in adult Lepidoptera are somewhat variable. Usually, the LPO of moths is about 100–300 μm deep and 30–80 μm wide. LPO sensilla can be divided into one to three morphological types. The number of LPO sensilla varies from 80 (Lee et al., 1985) to 1,750 (Kent et al., 1986) in different lepidopteran species.

Compared to antennae, the function of labial palps is largely unknown. At present, the most important function of labial palps in adult Lepidoptera that has been reported is detecting carbon dioxide (CO_2_). Electrophysiological recording preformed on the sensilla in the LPO of butterfly (Lee et al., 1985) and moth (Bogner et al., 1986; Stange et al., 1995; Guerenstein et al., 2004; Ning et al., 2016) all showed that the LPO sensilla react to CO_2_. CO_2_ is a ubiquitous source of ecologically relevant information in insect-plant interactions, insect-vertebrate interactions, and insect social behavior (Guerenstein and Hildebrand, 2008). Sensing CO_2_ is essential for foraging (Thom et al., 2004), mating (Choi et al., 2018), and oviposition (Myers et al., 1981; Stange, 1997) in many moth species of the Lepidoptera. These studies raised a general question about whether the LPO sensilla in lepidopteran species are sensitive to volatile compounds. Earlier report indicated that the LPO sensilla of *Rhodogastria* respond to cyclopentanone, acetic acid, octanol, limonene, citral, hexanal, butanal, and pentanal (Bogner et al., 1986), and the LPO sensilla in *Pieris brassicae* are responding to terpineol, cyclopentanone, cumol, acetic acid, propionic acid, and butyric acid (Bogner, 1990). According to the findings of these two articles, the labial palps in adult Lepidoptera that are excited by stimulation with CO_2_ may also respond to various volatile compounds. However, it is unknown whether these chemical odors elicited responses of labial palps in other species.

*Spodoptera frugiperda* (Lepidoptera: Noctuidae), also called fall armyworm, is native to America (Sparks, 1979) but has been spread to Africa (Goergen et al., 2016; Nagoshi et al., 2017; Stokstad, 2017), India (Ganiger et al., 2018), and China (Guo et al., 2018a; Li et al., 2019; Sun et al., 2019a,b). *S. frugiperda* has a wide host range of more than 350 species of plants, including corn, rice, wheat, soybean, and cotton (Montezano et al., 2018), and is one of the most damaging crop pests. There have been many latest investigations focusing on the management against this pest, such as genome editing of the receptor for *Bacillus thuringiensis* in *S. frugiperda* (Jin et al., 2019), the potential roles of *Junonia coenia* densovirus in *S. frugiperda* control (Chen et al., 2020), and the positive phototaxis of *S. frugiperda* (Liu et al., 2020). Elevated CO_2_ concentration was recently reported to affect the growth and development of *S. frugiperda* (Zhao et al., 2019), providing support for investigating the structure and function of the LPO, the CO_2_-sensitive organ. In this study, the distribution and fine structure of sensilla located in the LPO were investigated using scanning electron microscopy. Sensilla in the LPO were divided into two morphological types. Subsequently, we modified the electroantennogram (EAG) setup to function as the electrolabialpalpography (ELPG) to record the responses of labial palps to different concentrations of CO_2_ and 29 plant volatiles. Finally, the sensilla that responded to CO_2_ in the LPO were identified via the intracellular recording (ICR). The results indicated that there are two types of sensory neurons in the LPO of *S. frugiperda*, one of which could be strongly activated by different concentrations of CO_2_, while the other type showed no response to CO_2_. Our work established a foundation for further study of the roles of labial palps in insect olfaction-related behaviors. Based on these results, further investigations of the function of labial palps and their biological roles together with responses to CO_2_ and volatile ligands identified in this study of *S. frugiperda* are necessary.

## Materials and Methods

### Insects Rearing

The *S. frugiperda* colony was collected in the wild in Yunnan Province, China, in March, 2019, and then maintained at the Institute of Plant Protection, Chinese Academy of Agricultural Sciences, Beijing, China. The larvae were reared on an artificial diet and placed at 27 ± 1°C with a photoperiod of 14:10 h (L:D). Pupae were together kept in a gauze cage before eclosion. Adults were selected by sex and placed in separate test tubes after eclosion and fed 10% sugar solution every day. Adult females and males were used in all experiments.

### Light Microscopy and Biometry Measurements

The protruding head of adult *S. frugiperda* was fixed to the rim of a pipette tip by using dental wax and observed under a super-high magnification lens zoom 3D microscope (VHX-2000, Japan). The labial palps were dissected from the head using fine scissors. Scales covering the labial palps were cleared with double-sided tap. Then, the dehydrated and transparent labial palps were positioned on a microscopic slide with a drop of glycerin and a cover slip. Finally, the labial palps were observed and measured using a super-high magnification lens zoom 3D microscope (VHX-2000, Japan). We measured the length of each segment of labial palps, and the depth and diameter of the LPO.

### Scanning Electron Microscopy

The labial palps were removed from 3- to 4-day-old moths and then cleared with double-sided tape to remove the outer scales. In order to study the morphology of the sensilla in LPO, we split the LPO by using fine scissors. Next, these prepared samples were processed by a series of dehydration, drying, and last were sprayed with gold as described by Guo et al. (2018b). In the described steps, the critical point drier was LEICA EM CPD (Germany) and the type of a sputter-coating unit is EIKO IB-3 (Japan). Finally, the samples were investigated using a Hitachi SU8010 scanning electron microscope (Japan) at 10 kV.

### Electrolabialpalpography

Taking 3- to 4-day-old adult *S. frugiperda*, the labial palps were carefully cut from the base charily by using fine scissors, and surface scales were removed with double-sided tape. The treated labial palps were used for recording with the base inserted into the conducting gel (Parker Laboratories, United States) and the tip just contacting the conducting gel to ensure that the opening of the LPO, which harbors all the sensilla, was exposed to the air. The conducting gel was painted on the neutral arms of the metal electrode.

For CO_2_ stimulus, the mounted labial palp was excited with stimulus delivery in self-regulating stimulus flow controller, which was mainly comprised of a 3/2-way solenoid valve (XP-513, Japan) and two currents of equal flow rate at 0.8 L/min. One current called continuous flow was diverted through bottled synthetic air, and the other current called stimuli flow was diverted through bottled CO_2_ at different concentrations. Stimuli were provided for 1 s by controlling the 3/2-way solenoid valve and were delivered through a 14-cm-long metal tube. Commercially available compressed bottled CO_2_ gas stimuli were used at concentrations of 0.1, 1, and 10% (the remainder was synthetic air), and synthetic air was used as a control. To make synthetic air CO_2_-free, it contained only 21% O_2_ and 78% N_2._ All above gases in certificated gas cylinders were bought from company (Beijing Shangtonghong Chemical, China). The resulting ELPG amplitudes (negative potential) were recorded and analyzed by using EAG software (Syntech, Germany). The ELPG response values for CO_2_ were calculated by subtracting the value of the same labial palp corresponding to the blank control (synthetic air).

For odor stimuli, 10 μl of test solution or solvent was added in to filter paper strips (0.5 cm × 6 cm) inserted in a Pasteur pipette (15 cm long). A flow of purified and humidified air continuously blew toward the labial palp through a metal tube at 0.4 L/min. A stimulus air pulse was added for 200 ms. The intervals between two stimuli were 30 s. The Pasteur pipette connected to the stimulus air controller CS-55 (Syntech, Germany) was used for stimulation. The pre-amplifier was displayed on a computer via a software interface EAGPRO (Syntech, Germany), and action potentials were amplified, digitized, and visualized on a computer screen. The 29 chemical compounds (95% minimum purity compound) used in this study were purchased from Sigma-Aldrich (Germany). These compounds were dissolved in paraffin oil at the concentration of 1 μg/μl. For odor stimuli in ELPG assay, the recording of labial palp to paraffin oil was used as a control. The ELPG response values for odorants were calculated by subtracting the value of the same labial palp corresponding to the paraffin oil.

### Intracellular Recording

The 3- to 4-day-old female and male adults after emergence were wedged into a 1 ml plastic pipette tip with the narrow end cut to allow the head and the exposed labial palps to protrude. The protruding head and other organs, including antennae and proboscis, were all immobilized to the edge of the pipette tip with dental wax under a stereomicroscope, just leaving one of the labial palps accessible. The outer scales on the labial palp were removed carefully with double-sided adhesive tape, and then, the labial palp was fixed with dental wax to reveal just the tip of labial palp and the opening of the LPO.

Nerve impulses from single sensory neurons were recorded intracellularly using a sharp quartz electrode. Under a SZX16 microscope (Olympus, Japan), the reference electrode made of a silver wire was inserted into the moth eye, and the recording electrode which containing 0.2 M KAc was inserted vertically into the LPO via a micromanipulator (Leica, Germany). Spikes were recorded when the quartz electrode was inserted into a sensory neuron in sensilla. During the insertion of the recording electrode into the LPO, it was not possible to distinguish the different sensilla under the microscope because they are located inside the LPO and only the opening of LPO was visible. The amplified analog signals of the action potentials were captured and processed using a signal amplifier (Axoclamp 900A, United States) and a digital-to-analog converter (CED MICRO 1401, England). The recorded spikes activity was displayed on a computer screen using the software package Autospike 2 8.01 (Syntech, Germany).

For stimulus delivery, a 5 s CO_2_ stimulus flow was provided by a self-regulating stimulus flow controller. A flow of purified and humidified synthetic air (21% O_2_, 78% N_2_) was continuously blown on the opening of the LPO through a 14-cm-long metal tube by the self-regulating stimulus flow controller at 0.8 L/min. CO_2_ stimuli were represented at 0.1, 1, 10%, and synthetic air (21% O_2_ and 78% N_2_) was used as a control. The response values to specific concentration of CO_2_ were calculated using the formula: T − C, where T represents the differences in spike numbers observed between 5 s before and 5 s after CO_2_ delivery, and C represents the differences in spike numbers observed between 5 s before and 5 s after control (synthetic air) delivery.

### Image Processing and Statistical Analysis

The classification and naming of sensilla in LPO were described in Zhao et al. (2013). ELPG statistics and graphing were performed using GraphPad Prism. The measured data of labial palps were analyzed in Microsoft Office Excel 2007. LPO sensilla were measured by LSM Image Browser and analyzed in Microsoft Office Excel 2007. Differences in the response value (or measured data) of females and males were analyzed by *t*-test. Spikes separated from noise were analyzed and evaluated by the computer software Autospike (Syntech, Germany).

## Results

### Morphological Structure of the Labial Palp and LPO in Adult *S. frugiperda*

**Figure 1:**
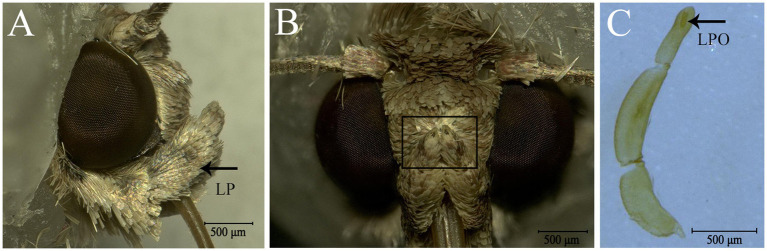
Labial palps (LP) and labial-palp pit organ (LPO) of *Spodoptera frugiperda.* **(A)** Lateral view of the head and the right LP (black arrow); the LPs are covered densely with scales and are located on each side of the proboscis just below the eyes. **(B)** Front view of the head and the LPs. The black boxed area shows the tip of the two LPs and the opening of the LPO. **(C)** Three segments of the LP; the black arrow shows the LPO.

**Figure 2:**
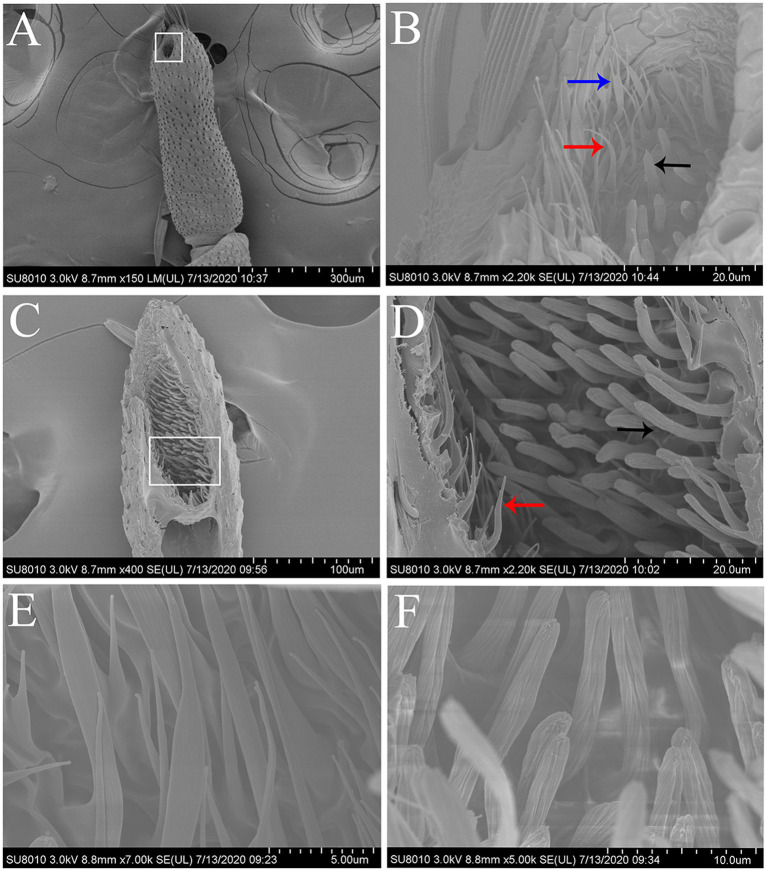
Scanning electron micrographs of the terminal segment of labial palps and the LPO sensilla in *S. frugiperda.* **(A)** The terminal segment of the labial palp showing the opening of the LPO. White boxed area: opening of the LPO. **(B)** Scanning electron micrograph of the sensilla located around the opening of the LPO, which contains hair-shaped sensilla (red and blue arrows) and club-shaped sensilla (black arrow). Two hair-shaped sensilla subtypes were found hair-shaped sensilla (red arrow) and hair-shaped sensilla with forked tips (blue arrow). **(C)** Longitudinal section of the LPO in one labial palp showing two main types of sensilla. **(D)** Enlarged image of white boxed area in **(C)**. The LPO has two types of sensilla, i.e., hair-shaped sensilla (red arrow) and club-shaped sensilla (black arrow). These two types of sensilla are distributed in separate areas along the vertical axis of the LPO. **(E)** Scanning electron micrograph of hair-shaped sensilla. **(F)** Scanning electron micrograph of club-shaped sensilla.

Adults of *S. frugiperda* possess a pair of labial palps located on the ventral side of the head that enfold the proboscis (Figure 1A). The two labial palps are entirely covered by dense scales and have two small holes at the top (Figure 1B). When the scales are removed, each labial palp contains three segments and is tubular (Figure 1C). Each segment of labial palps in *S. frugiperda* differs in the morphological structure and length (Figure 1C; Table 1). The first segment of the labial palp, which is connected to the head, is about 675 μm long, and the second segment is about 857 μm. The third segment is about 478 μm long. An opening near the tip of the third segment extends to a cavity called the LPO (Figures 1C, 2A), which is about 117 μm deep and of variable diameter (Table 1). In females, the diameter of the LPO opening is 43.08 ± 1.43 μm (mean ± SE, *n* = 13). In males, the diameter of the LPO opening is 39.15 ± 1.03 μm (mean ± SE, *n* = 20). The diameter of the LPO opening in females is significantly longer than in males. The inner diameter of the LPO at the midpoint is approximately 38 μm, and the inner diameter of the LPO at the base is about 33 μm.

**Table 1:** The length of each segment of labial palp (LP) and the depth and diameters of LPO of *S. frugiperda*.

	Female	Male	*t*-test
Length of the first LP segment (μm)	688.26 ± 16.30 (23)	663.39 ± 11.03 (23)	*p =* 0.213
Length of the second LP segment (μm)	869.57 ± 17.80 (23)	846.04 ± 11.00 (23)	*p =* 0.267
Length of the third LP segment (μm)	470.78 ± 12.04 (23)	486.43 ± 10.27 (23)	*p* = 0.328
Depth of LPO (μm)	118.35 ± 2.60 (17)	116.22 ± 2.09 (23)	*p =* 0.521
Diameter of LPO opening (μm)	43.08 ± 1.43 (13)	39.15 ± 1.03 (20)	*p =* 0.029
Inner diameter of LPO at half length (μm)	38.77 ± 1.43 (13)	37.53 ± 0.74 (20)	*p =* 0.473
Inner diameter of LPO at the base (μm)	33.69 ± 1.22 (13)	32.80 ± 1.08 (20)	*p =* 0.595

Data in the table are means ± SE. The numbers in parentheses indicate the replicates of measurement.

### Fine Morphological Structure of Sensilla Located in LPO

The LPO is densely packed with approximately 300 sensilla, which comprise hair-shaped sensilla and club-shaped sensilla (Figure 2C). The hair-shaped sensilla are slender, and the ends are slightly bent (Figures 2B,D,E). Some hair-shaped sensilla have forked tips (Figure 2B; blue arrow). The club-shaped sensilla are short and rod-like, and their surfaces have grooves (Figures 2D,F). Hair-shaped sensilla and club-shaped sensilla are distributed in separate areas along the vertical axis of the LPO (Figure 2D). The length and basal diameter of each sensillum category are shown in Table 2. In females, the hair-shaped sensilla are 23.92 ± 0.58 μm long (mean ± SE, *n* = 5) and the basal diameters are 2.74 ± 0.25 μm (mean ± SE, *n* = 5), while the club-shaped sensilla are 13.10 ± 0.54 μm long (mean ± SE, *n* = 5) and the basal diameters are 2.02 ± 0.06 μm long (mean ± SE, *n* = 5). In males, the hair-shaped sensilla are 25.44 ± 0.50 μm long (mean ± SE, *n* = 14) and the basal diameters are 2.88 ± 0.15 μm (mean ± SE, *n* = 4), while the club-shaped sensilla are 12.80 ± 0.27 μm long (mean ± SE, *n* = 14) and the basal diameters are 2.08 ± 0.03 μm (mean ± SE, *n* = 14). The *t*-test results show no significant difference in the size of each sensillum type between females and males (Table 2).

**Table 2:** The length of LPO sensilla in *S. frugiperda* and their diameter at the base.

	Female	Male	*t*-test
Length of hair-shaped sensilla (μm)	23.92 ± 0.58 (5)	25.44 ± 0.50 (14)	*p =* 0.058
Basal diameter of hair-shaped sensilla (μm)	2.74 ± 0.11 (5)	2.87 ± 0.15 (14)	*p =* 0.48
Length of club-shaped sensilla (μm)	13.10 ± 0.54 (5)	12.79 ± 0.27 (14)	*p =* 0.46
Basal diameter of club-shaped sensilla	2.02 ± 0.06 (5)	2.07 ± 0.03 (14)	*p =* 0.37

Data in the table are means ± SE. The numbers in parentheses indicate the replicates of measurement.

### ELPG Response of Labial Palp to CO_2_ and Plant Volatiles

**Figure 3:**
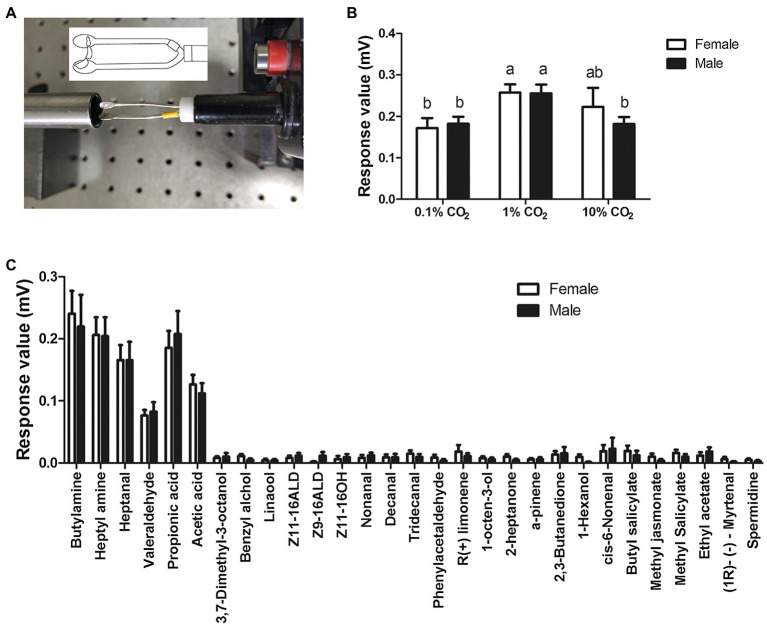
Electrolabialpalpography (ELPG) response of labial palps in *S. frugiperda* to CO_2_ and plant volatiles. **(A)** Schematic showing a labial palp mounted on the electrode in the ELPG assay. **(B)** ELPG response of labial palp in *S. frugiperda* to different concentrations of CO_2_ including 0.1, 1, and 10%. Data are the mean ± SD (*n* = 30). **(C)** ELPG responses of labial palp in *S. frugiperda* to 29 plant volatiles. Data are the mean ± SE (*n* = 15).

In order to demonstrate the labial palps of *S. frugiperda* also response to odor stimulation besides CO_2_ stimulation, we performed ELPG on the female and male labial palps (Figure 3A). The labial palp in *S. frugiperda* adults responded obviously to different concentrations of CO_2_. The magnitude of response mainly depended on the concentration of CO_2_, with the strongest responses to stimulus of 1% CO_2_, at about 0.26 ± 0.02 mV (mean ± SE, *n* = 30), and the weakest responses to the stimulus of 0.1% CO_2_, at about 0.18 ± 0.02 mV (mean ± SE, *n* = 30; Figure 3B). In females, the responses of labial palp to 1% CO_2_ are significantly greater than to 0.1% CO_2_. There is no significant difference in the responses of labial palp to 1% CO_2_ and 10% CO_2_, and 0.1% CO_2_ and 10% CO_2_. In males, the responses of labial palp to 1% CO_2_ is significantly greater than to 0.1% CO_2_ and is significantly less than to 10% CO_2_. There is no significant difference in the responses of labial palp to 0.1% CO_2_ and 10% CO_2_ (Figure 3B). However, The response value of labial palp to the same concentration of CO_2_ was not significantly different between females and males (Figure 3B).

To verify whether the labial palp, as an olfactory organ, responded to volatile compounds other than CO_2_, we also investigated the electrophysiological responses of labial palps to 29 volatile compounds (Figure 3C). The labial palps of *S. frugiperda* obviously responded to six compounds: butylamine, heptylamine, heptanal, valeraldehyde, propionic acid, and acetic acid (Figure 3C). As with CO_2_, responses were not significantly different between females and males.

### Recording From LPO Sensilla to CO_2_

**Figure 4:**
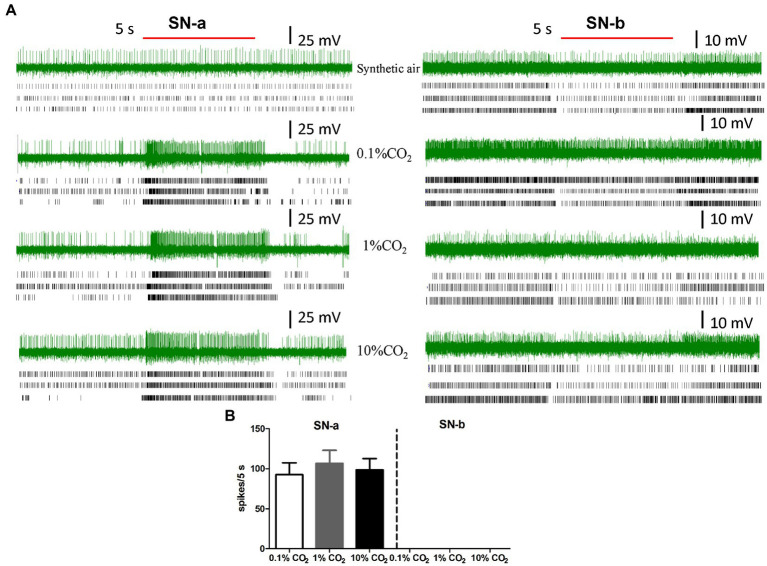
Intracellular recording (ICR) analysis of the sensory neurons housed in LPO sensilla. **(A)** The representative traces of ICRs, three example of rater plots of action potentials of responses of sensory neurons in SN-a and SN-b to synthetic air, 0.1% CO_2_, 1% CO_2_, and 10% CO_2_. The red bold line represents the 5 s stimulation. The letters (SN-a, SN-b) represent the different types of sensory neurons in LPO sensilla. **(B)** Quantification of the mean responses of SN-a and SN-b in LPO sensilla to 0.1% CO_2_, 1% CO_2_, and 10% CO_2_. Data are the mean ± SE (*n* = 10–16). The response values to specific concentration of CO_2_ were calculated using the formula: T − C, where T represents the differences in spike numbers observed between 5 s before and 5 s after CO_2_ delivery, and C represents the differences in spike numbers observed between 5 s before and 5 s after control (synthetic air) delivery.

In order to check the existence of sensilla in the LPO that respond to CO_2_, we performed ICR on sensory neurons in LPO sensilla from male and female labial palps. CO_2_-sensitive neurons were found in LPO sensilla of adult *S. frugiperda* (Figure 4; SN-a). We also found sensory neurons that did not respond to CO_2_ in the LPO sensilla (Figure 4; SN-b). The sensory neurons that responded to CO_2_ were labeled sensory neuron a (SN-a), while those that did not respond to CO_2_ were labeled sensory neuron b (SN-b; Figure 4). We successfully recorded 11 adults *S. frugiperda* in total, including six females and five males. A total of 22 neurons with unambiguous spikes from eight insects (four females and four males) were analyzed. For SN-a, there was a strong excitatory response to CO_2_ stimulus at concentrations of 0.1, 1, and 10% (Figure 4A) and the mean activated spikes of these neurons were, respectively, about 93 spikes/5 s, 107 spikes/5 s, and 99 spikes/5 s (Figure 4B). Besides, the responses of SN-a were not significantly different between these three concentrations of CO_2_.

## Discussion

Structure characterization of an olfactory organ and its sensilla are vital to understand how the olfactory organ performs its ecological function. This model has been widely used in the surveys of antennae in lepidopteran insects. In an effort to research the function of another crucial olfactory organ, the labial palp, the fine structure of LPO and LPO sensilla in *S. frugiperda* were investigated in detailed in the present study. For the structure of LPO, we found a significant difference in the diameter of the LPO opening between females and males in *S. frugiperda*. The diameter of the LPO opening in *S. frugiperda* exhibited distinct sexual dimorphism and was much longer in females (43.08 μm) than in males (39.15 μm). In other reported Noctuidae species (Zhao et al., 2013; Dong et al., 2014), the diameter of the LPO opening tends to be the same size in both sexes. The sexual dimorphism of the diameter of the LPO opening is described for the first time in this study, although sexual dimorphism also occurs in the length of labial palps in *Cactoblastis cactorum* (Stange et al., 1995), *Mythimna separata* (Dong et al., 2014), *Carposina sasakii* (Chen and Hua, 2016), and *Plutella xylostella* (Yan et al., 2019). This phenomenon may be related to sex-specific differences in behavior, such as courtship and oviposition. For example, *C. cactorum* probes the surface of a plant with their labial palps before ovipositing, so the length of labial palps in females is much longer than in males (Stange et al., 1995). The differences between the sexes in the diameter of the LPO opening may also be due to the ovipositing behavior of female *S. frugiperda*, though further studies are required to confirm this.

In our study, the densely packed array of LPO sensilla in *S. frugiperda* can be divided into two morphological types: hair-shaped sensilla and club-shaped sensilla, like that in *C. cactorum* (Stange et al., 1995), *Helicoverpa armigera* (Zhao et al., 2013), *M. separata* (Dong et al., 2014), and *C. sasakii* (Chen and Hua, 2016); hair-shaped sensilla in *C. cactorum* and *C. sasakii* have not been described in detail. However, there is only one kind of LPO sensilla in some moth species. For example, in *Rhodogastria* spp. the LPO is densely packed with smooth-walled sensilla of uniform appearance (Bogner et al., 1986), and the LPO in *Plodia interpunctella* also contains a single small trichoid sensillum (Barcaba and Krenn, 2015). The LPO in *Grapholita molesta* (Song et al., 2016) contains three categories of sensilla: hair-shaped sensilla, club-shaped sensilla, and small mastoid sensilla. Although the categories of LPO sensilla are identical, the hair-shaped sensilla and club-shaped sensilla in the LPO of *S. frugiperda* are distributed in separate areas along the vertical axis of LPO, whereas they are situated in the upper half and the lower half of the pit in *H. armigera* (Zhao et al., 2013) and *M. separata* (Dong et al., 2014). This type of distribution of LPO sensilla in *S. frugiperda* is described for the first time. In summary, the differences of LPO sensilla in categories and location may be dependent on the insect species, or related to the behavior of insects and the function of the labial palps.

Electrolabialpalpography and ICR data in the present investigation support the idea that *S. frugiperda* have CO_2_-sensitive neurons in the LPO, as reported in other lepidopteran species (Bogner et al., 1986; Stange et al., 1995; Stange, 1997; Guerenstein et al., 2004; Ning et al., 2016). This suggests that CO_2_-detection is a universal function of the LPO in Lepidoptera. Interestingly, we found a kind of sensory neuron that was non-responsive to CO_2_ in the LPO of *S. frugiperda*, which has never been reported before. This finding implies that LPO sensilla are not uniform in detecting CO_2_ and they may also respond to other odorants. Our electrophysiological recording results strongly support the hypothesis that LPO sensilla can respond to volatile chemicals. The labial palps of *S. frugiperda* obviously responded to six of 29 volatiles tested in our experiment, including butylamine, heptylamine, heptanal, valeraldehyde, propionic acid, and acetic acid. Electrophysiologically active compounds in this study, propionic acid and acetic acid, which are volatiles from host plants, have been reported in other lepidopteran insects (Bogner et al., 1986; Bogner, 1990). In addition, several kinds of odorants and their analogues found to be effective stimuli in *S. frugiperda* also activate CO_2_ receptors of antennae in flies and CO_2_ receptors of maxillary palps in mosquitoes (Turner and Ray, 2009; Tauxe et al., 2013; Macwilliam et al., 2018). The class of odorants also present in ripe fruits has important ecological significance, as they can modify the CO_2_ avoidance behavior, helping the host-seeking behavior of *Drosophila melanogaster* (Turner and Ray, 2009). Hence, we predict that olfactory perception of ecologically relevant volatiles occurs on labial palps of *S. frugiperda*, but its role in behaviors remains to be investigated.

We speculate that the gustatory receptor (GR) genes and ionotropic receptor (IR) genes have pivotal roles in detecting CO_2_ and other volatile compounds for *S. frugiperda* LPO. Two GRs, GR21a and GR63a, were identified as the CO_2_ receptor genes for the first time in *D. melanogaster* (Jones et al., 2007; Kwon et al., 2007). Later, their homologous genes, GR1, GR2, and GR3, were successively identified as the CO_2_ receptors in many mosquito species (Kent et al., 2008; Robertson and Kent, 2009; Coutinho-Abreu et al., 2019). GR1, GR2, and GR3, which are highly expressed in labial palps, have been identified using phylogenetic analysis in several lepidopteran species (Briscoe, 2000; Spaethe and Briscoe, 2004; Liu et al., 2014; Xu and Anderson, 2015; Zhang et al., 2015; Liu et al., 2017), and their functions have been confirmed (Xu and Anderson, 2015; Ning et al., 2016). These three GRs are likely required for CO_2_ detection in *S. frugiperda*. For volatile compounds detection, the molecular mechanism is generally related to odorant receptors (ORs). However, these six odors, which excited labial palps of *S. frugiperda*, mainly contain acid, aldehyde, and amine. It has been reported that sensing of this class of odors was involved in IRs predominantly (Zhang and Wang, 2020). Moreover, there were indeed IRs identified in labial palps of the lepidopteran *H. armigera* (Guo et al., 2018b). Analogously, IRs might also be the receptor for detecting these six odors in *S. frugiperda*.

Our exploration of ultrastructural characteristics of LPO sensilla and their physiological functions in *S. frugiperda* might be useful not only for obtaining knowledge about the function of labial palps but also for controlling this serious insect pest. Further study is needed to clarify the physiological functions of the two morphological types of sensilla in LPO, hair-shaped sensilla and club-shaped sensilla, and confirm that these two types of LPO sensilla in *S. frugiperda* are separately sensitive to CO_2_ and airborne chemicals. Further behavioral studies and molecular investigations of the labial palps are necessary to better understand the ecological significance and molecular basis of olfaction in *S. frugiperda*.

## Data Availability Statement

The original contributions presented in the study are included in the article/supplementary material, and further inquiries can be directed to the corresponding author.

## Author Contributions

JS and GW designed the experiments. QC, XL, SC, BM, and MG performed the experiments. QC and GW wrote the manuscript and analyzed the data. JS and GW revised the manuscript. All authors contributed to the article and approved the submitted version.

## Conflict of Interest

The authors declare that the research was conducted in the absence of any commercial or financial relationships that could be construed as a potential conflict of interest.

## Publisher’s Note

All claims expressed in this article are solely those of the authors and do not necessarily represent those of their affiliated organizations, or those of the publisher, the editors and the reviewers. Any product that may be evaluated in this article, or claim that may be made by its manufacturer, is not guaranteed or endorsed by the publisher.

## References

[ref1] Barcaba T. Krenn H. W. (2015). The mouthparts of adult Indian meal moths, *Plodia interpunctella* (Hübner, 1813) (Lepidoptera: Pyralidae). Entomol. Aust. 22, 91–105.

[ref2] Bogner F. (1990). Sensory physiological investigation of carbon dioxide receptors in Lepidoptera. J. Insect Physiol. 36, 951–957. 10.1016/0022-1910(90)90083-R

[ref3] Bogner F. Boppré M. Ernst K. D. Boeckh J. (1986). CO_2_ sensitive receptors on labial palps of *Rhodogastria* moths (Lepidoptera: Arctiidae): physiology, fine structure and central projection. J. Comp. Physiol. A 158, 741–749. 10.1007/BF01324818, PMID: 3090241

[ref4] Bohbot J. D. Sparks J. T. Dickens J. C. (2014). The maxillary palp of *Aedes aegypti*, a model of multisensory integration. Insect Biochem. Mol. Biol. 48, 29–39. 10.1016/j.ibmb.2014.02.007, PMID: 24613607

[ref5] Briscoe A. D. (2000). Six opsins from the butterfly *Papilio glaucus*: molecular phylogenetic evidence for paralogous origins of red-sensitive visual pigments in insects. J. Mol. Evol. 51, 110–121. 10.1007/s002390010071, PMID: 10948267

[ref6] Chen J. Hua B. (2016). Sexual dimorphism of adult labial palps of the peach fruit moth *Carposina sasakii* Matsumura (Lepidoptera: Carposinidae) with notes on their sensilla. Acta Zool. 97, 42–48. 10.1111/azo.12103

[ref7] Chen Z. W. Yang Y. C. Zhang J. F. Jin M. H. Xiao Y. T. Xia Z. C.. (2020). Susceptibility and tissue specificity of *Spodoptera frugiperda* to *Junonia coenia* densovirus. J. Integr. Agr. 20, 840–849. 10.1016/S2095-3119(20)63163-X

[ref8] Choi K. S. Ahn S. J. Kim S. B. Ahn J. J. Jung B. N. Go S. W.. (2018). Elevated CO_2_ may alter pheromonal communication in *Helicoverpa armigera* (Lepidoptera: Noctuidae). Physiol. Entomol. 43, 169–179. 10.1111/phen.12239

[ref9] Coutinho-Abreu L. V. Sharma K. Cui L. Yan G. Ray A. (2019). Odorant ligands for the CO_2_ receptor in two *Anopheles* vectors of malaria. Sci. Rep. 9:2549. 10.1038/s41598-019-39099-0, PMID: 30796292 PMC6385339

[ref10] Dong J. Liu H. Tang Q. Liu Y. Zhao X. Wang G. (2014). Morphology, type and distribution of the labial-palp pit organ and its sensilla in the oriental armyworm, *Mythimna separata* (Lepidoptera: Noctuidae). Acta Entomol. Sin. 57, 681–687. 10.16380/j.kcxb.2014.06.012

[ref11] Faucheux M. J. (2008). Mouthparts and associated sensilla of a South American moth *Synempora andesae* (Lepidoptera: Neopseustidae). Rev. Soc. Entomol. Argent. 67, 21–33. 10.1590/S0373-56802008000100003

[ref12] Galizia C. G. Rossler W. (2010). Parallel olfactory systems in insects: anatomy and function. Annu. Rev. Entomol. 55, 399–420. 10.1146/annurev-ento-112408-085442, PMID: 19737085

[ref13] Ganiger P. C. Yeshwanth H. M. Muralimohan K. Vinay N. Kumar A. R. V. Chandrashekara K. (2018). Occurrence of the new invasive pest, fall armyworm, *Spodoptera frugiperda* (JE Smith) (Lepidoptera: Noctuidae), in the maize fields of Karnataka, India. Curr. Sci. 115, 621–623. 10.18520/cs/v115/i4/621-623

[ref14] Goergen G. Kumar P. L. Sankung S. B. Togola A. Tamo M. (2016). First report of outbreaks of the fall armyworm *Spodoptera frugiperda* (J E Smith) (Lepidoptera, Noctuidae), a new alien invasive pest in west and central Africa. PLoS One 11:e0165632. 10.1371/journal.pone.0165632, PMID: 27788251 PMC5082806

[ref15] Guerenstein P. G. Christensen T. A. Hildebrand J. G. (2004). Sensory processing of ambient CO_2_ information in the brain of the moth *Manduca sexta*. J. Comp. Physiol. A 190, 707–725. 10.1007/s00359-004-0529-0 15235811

[ref16] Guerenstein P. G. Hildebrand J. G. (2008). Roles and effects of environmental carbon dioxide in insect life. Annu. Rev. Entomol. 53, 161–178. 10.1146/annurev.ento.53.103106.093402, PMID: 17803457

[ref17] Guo J. F. Zhao J. Z. He K. L. Zhang F. Wang Z. Y. (2018a). Potential invasion of the crop-devastating insect pest fall armyworm *Spodoptera frugiperda* to China. Plant Prot. 44, 1–10. 10.16688/j.zwbh.2018452

[ref18] Guo M. B. Chen Q. Y. Liu Y. Wang G. R. Han Z. J. (2018b). Chemoreception of mouthparts: sensilla morphology and discovery of chemosensory genes in proboscis and labial palps of adult *Helicoverpa armigera* (Lepidoptera: Noctuidae). Front. Physiol. 9:970. 10.3389/fphys.2018.00970 30131703 PMC6091246

[ref19] Jin M. H. Tao J. H. Li Q. Cheng Y. Sun X. X. Wu K. M.. (2019). Genome editing of the *SfABCC2* gene confers resistance to *Cry1F* toxin from *Bacillus thuringiensis* in *Spodoptera frugiperda*. J. Integr. Agr. 20, 815–820. 10.1016/S2095-3119(19)62772-3

[ref20] Jones W. D. Cayirlioglu P. Kadow I. G. Vosshall L. B. (2007). Two chemosensory receptors together mediate carbon dioxide detection in *Drosophila*. Nature 445, 86–90. 10.1038/nature05466, PMID: 17167414

[ref21] Kent K. S. Harrow I. D. Quartararo P. Hildebrand J. G. (1986). An accessory olfactory pathway in Lepidoptera: the labial pit organ and its central projections in *Manduca sexta* and certain other sphinx moths and silk moths. Cell Tissue Res. 245, 237–245. 10.1007/BF00213927, PMID: 3742559

[ref22] Kent L. B. Walden K. K. Robertson H. M. (2008). The Gr family of candidate gustatory and olfactory receptors in the yellow-fever mosquito *Aedes aegypti*. Chem. Senses 33, 79–93. 10.1093/chemse/bjm067, PMID: 17928357

[ref23] Kwon J. Y. Dahanukar A. Weiss L. A. Carlson J. R. (2007). The molecular basis of CO_2_ reception in *Drosophila*. Proc. Natl. Acad. Sci. 104, 3574–3578. 10.1073/pnas.0700079104, PMID: 17360684 PMC1805529

[ref24] Lee J. K. Selzer R. Altner H. (1985). Lamellated outer dendritic segments of a chemoreceptor within wall-pore sensilla in the labial palp-pit organ of the butterfly, *Pieris rapae* L. (Insecta, Lepidoptera). Cell Tissue Res. 240, 333–342. 10.1007/BF00222343

[ref25] Li G. P. Ji T. J. Sun X. X. Jiang Y. Y. Wu K. M. Feng H. Q. (2019). Susceptibility evaluation of invaded *Spodoptera frugiperda* population in Yunnan province to five *Bt* toxins. Plant Prot. 45, 15–20. 10.16688/j.zwbh.2019201

[ref26] Liu N. Y. Xu W. Papanicolaou A. Dong S. L. Anderson A. (2014). Identification and characterization of three chemosensory receptor families in the cotton bollworm *Helicoverpa armigera*. BMC Genomics 15:597. 10.1186/1471-2164-15-597, PMID: 25027790 PMC4112213

[ref27] Liu Y. J. Zhang D. D. Yang L. Y. Dong Y. H. Liang G. M. Donkersley P.. (2020). Analysis of phototactic responses in *Spodoptera frugiperda* using *Helicoverpa armigera* as control. J. Integr. Agr. 20, 821–828. 10.1016/S2095-3119(19)62863-7, PMID: 25027790

[ref28] Liu Z. Wang X. Lei C. Zhu F. (2017). Sensory genes identification with head transcriptome of the migratory armyworm *Mythimna separata*. Sci. Rep. 7:46033. 10.1038/srep46033, PMID: 28387246 PMC5384095

[ref29] Macwilliam D. Kowalewski J. Kumar A. Pontrello C. Ray A. (2018). Signaling mode of the broad-spectrum conserved CO_2_ receptor is one of the important determinants of odor valence in *Drosophila*. Neuron 97, 1153.e4–1167.e4. 10.1016/j.neuron.2018.01.028, PMID: 29429938 PMC5871350

[ref30] Montezano D. G. Specht A. Sosa-Gómez D. R. Roque-Specht V. F. Sousa-Silva J. C. Paula-Moraes S. V.. (2018). Host plants of *Spodoptera frugiperda* (Lepidoptera: Noctuidae) in the Americas. Afr. Entomol. 26, 286–300. 10.4001/003.026.0286

[ref31] Myers J. H. Monro J. Murray N. (1981). Egg clumping, host plant selection and population regulation in *Cactoblastis cactorum* (Lepidoptera). Oecologia 51, 7–13. 10.1007/BF00344644, PMID: 28310301

[ref32] Nagoshi R. N. Koffi D. Agboka K. Tounou K. A. Banerjee R. Jurat-Fuentes J. L.. (2017). Comparative molecular analyses of invasive fall armyworm in Togo reveal strong similarities to populations from the eastern United States and the Greater Antilles. PLoS One 12:e0181982. 10.1371/journal.pone.0181982, PMID: 28738081 PMC5524310

[ref33] Ning C. Yang K. Xu M. Huang L. Q. Wang C. Z. (2016). Functional validation of the carbon dioxide receptor in labial palps of *Helicoverpa armigera* moths. Insect Biochem. Mol. Biol. 73, 12–19. 10.1016/j.ibmb.2016.04.002, PMID: 27060445

[ref34] Robertson H. M. Kent L. B. (2009). Evolution of the gene lineage encoding the carbon dioxide receptor in insects. J. Insect Sci. 9:19. 10.1673/031.009.1901, PMID: 19613462 PMC3011840

[ref35] Song Y. Q. Sun H. Z. Wu J. X. (2016). Ultrastructural characteristics of the proboscis and the labial palp pit organ in the oriental fruit moth, *Grapholita molesta*. Bull. Insectol. 69, 59–66.

[ref36] Spaethe J. Briscoe A. D. (2004). Early duplication and functional diversification of the opsin gene family in insects. Mol. Biol. Evol. 21, 1583–1594. 10.1093/molbev/msh162, PMID: 15155799

[ref37] Sparks A. N. (1979). A review of the biology of the fall armyworm. Fla. Entomol. 62, 82–87. 10.2307/3494083

[ref38] Stange G. (1997). Effects of changes in atmospheric carbon dioxide on the location of hosts by the moth, *Cactoblastis cactorum*. Oecologia 110, 539–545. 10.1007/s004420050192, PMID: 28307247

[ref39] Stange G. Monro J. Stowe S. Osmond C. B. (1995). The CO_2_ sense of the moth *Cactoblastis cactorum* and its probable role in the biological control of the CAM plant *Opuntia stricta*. Oecologia 102, 341–352. 10.1007/BF00329801, PMID: 28306845

[ref40] Stange G. Stowe S. (1999). Carbon-dioxide sensing structures in terrestrial arthropods. Microsc. Res. Tech. 47, 416–427. 10.1002/(SICI)1097-0029(19991215)47:6<416::AID-JEMT5>3.0.CO;2-X, PMID: 10607381

[ref41] Stokstad E. (2017). New crop pest takes Africa at lightning speed. Science 356, 473–474. 10.1126/science.356.6337.473, PMID: 28473543

[ref42] Sun X. X. Hu C. X. Jia H. R. Wu Q. L. Shen X. J. Zhao S. Y.. (2019a). Case study on the first immigration of fall armyworm *Spodoptera frugiperda* invading into China. J. Integr. Agr. 20, 664–672. 10.1016/S2095-3119(19)62839-X

[ref43] Sun X. X. Zhao S. Y. Jin M. H. Zhao H. Y. Li G. P. Zhang H. W.. (2019b). Larval spatial distribution pattern and sampling technique of the fall armyworm *Spodoptera frugiperda* in maize fields. Plant Prot. 45, 13–18. 10.16688/j.zwbh.2019115

[ref44] Syed Z. Leal W. S. (2007). Maxillary palps are broad spectrum odorant detectors in *Culex quinquefasciatus*. Chem. Senses 32, 727–738. 10.1093/chemse/bjm040, PMID: 17569743

[ref45] Tauxe G. M. Macwilliam D. Boyle S. M. Guda T. Ray A. (2013). Targeting a dual detector of skin and CO_2_ to modify mosquito host seeking. Cell 155, 1365–1379. 10.1016/j.cell.2013.11.013, PMID: 24315103 PMC3899525

[ref46] Thom C. Guerenstein P. G. Mechaber W. L. Hildebrand J. G. (2004). Floral CO_2_ reveals flower profitability to moths. J. Chem. Ecol. 30, 1285–1288. 10.1023/B:JOEC.0000030298.77377.7d, PMID: 15303329

[ref47] Turner S. L. Ray A. (2009). Modification of CO_2_ avoidance behaviour in *Drosophila* by inhibitory odorants. Nature 461, 277–281. 10.1038/nature08295, PMID: 19710651

[ref48] Xu W. Anderson A. (2015). Carbon dioxide receptor genes in cotton bollworm *Helicoverpa armigera*. Sci. Nat. 102:11. 10.1007/s00114-015-1260-0, PMID: 25724420

[ref49] Yan X. Z. Wang Z. Y. Duan Y. Feng X. M. Deng C. P. Wu A. H.. (2019). Ultrastructure of sensilla on labial palps and the central projection of their sensory neurons in *Plutella xylostella* (Lepidoptera: Plutellidae) adults. Acta Entomol. Sin. 62, 205–214. 10.16380/j.kcxb.2019.02.007

[ref50] Zhang J. Wang B. Dong S. Cao D. Dong J. Walker W. B.. (2015). Antennal transcriptome analysis and comparison of chemosensory gene families in two closely related noctuidae moths, *Helicoverpa armigera* and *H. assulta*. PLoS One 10:e0117054. 10.1371/journal.pone.0117054, PMID: 25659090 PMC4319919

[ref51] Zhang X. X. Wang G. R. (2020). Advances in research on the identification and function of ionotropic receptors in insects. Chinese J. Appl. Entomol. 57, 1046–1055. 10.7679/j.issn.2095-1353.2020.105

[ref52] Zhao W. J. He S. Q. Lu Z. H. Liu M. R. Ma L. Q. Wang J.. (2019). Direct effects of elevated CO_2_ concentration on development of fall armyworm *Spodoptera frugiperda* (J. E. Smith). J. Environ. Ent. 41, 736–741. 10.3969/j.issn.1674-0858.2019.04.7

[ref53] Zhao X. C. Tang Q. B. Berg B. G. Liu Y. Wang Y. R. Yan F. M.. (2013). Fine structure and primary sensory projections of sensilla located in the labial-palp pit organ of *Helicoverpa armigera* (Insecta). Cell Tissue Res. 282, 237–249. 10.1007/BF00319115, PMID: 23736380

